# Diagnostic and Practical Essays on Medical and Surgical Subjects

**Published:** 1839-10

**Authors:** 


### ART. VIII.

1. *Diagnostisch-praktische Abhandlungen aus dem Gebiete der Medicin und Chirurgie; durch Krankheitsfälle erläutert.* Von Dr. LÖWENHARDT. Zweiter Theil.—*Prenzlau*, 1838. 8vo, pp. 425.

*Diagnostic and practical Essays on Medical and Surgical Subjects; illustrated by cases.* By Dr. LÖWENHARDT. Part. II.—*Prenzlau*, 1838.

2. *Versuche für die praktische Heilkunde.* Von FERDINAND JAHN. Erste Heft.—*Eisenach*, 1835. 8vo, pp. 216.

*Essays on Practical Medicine.* By FERDINAND JAHN. Part I.—*Eisenach*, 1835.

WE join these works together because they are composed on the same plan and have the same object of conveying information in practical medicine. As they are also both productions of eminent practitioners, they may serve as illustrations of the actual pathology and therapeutics of our brethren in Germany. We shall notice them separately.

I. In the Fourth Number of our Journal we noticed the first part of Dr. Löwenhardt's essays, and especially directed the attention of our readers to a very valuable paper on inflammation of the ovaries. The contents of the present volume are more diversified than those of its predecessor, and all of the papers present, amid some superfluity of words and trifling details, so much interesting matter, that we will endeavour to lay a short abstract of them before our readers.

Dr. Löwenhardt commences his first essay—*On the employment of large mercurial inunctions in various diseases*—by some general observations on the use of mercurial ointment. He is in the practice of directing from two to four drachms of the unguentum hydrargyri to be rubbed in every two, sometimes every four hours, until a gentle salivation is produced. The production of profuse ptyalism was never aimed at, nor does it appear to have often occurred; but two cases of fatal mercurial eczema are related, one of which took place in Dr. Löwenhardt's practice, and the other was met with in a young man who had made a few applications of mercurial ointment to the pubes, in order to destroy pediculi. After the preliminary remarks, the author proceeds to describe the diseases in which he has reaped benefit from this method of introducing mercury into the system.

The fatal termination of many cases of *apoplexia sanguinea,* in which the patient gradually sinks into a state of coma, whence all efforts to rouse him are unavailing, induced Dr. L. to make a trial of mercurial inunction. One instance is related in which venesection was performed, stimulant enemata were given, and the usual remedies were employed during twenty-four hours without any improvement in the patient's condition, and in which subsequent inunction of three ounces of mercurial ointment, producing salivation, was followed by amendment of all the symptoms, and ultimate recovery. The second case is still more striking. It occurred in a man seventy-eight years old who had already experienced two apoplectic seizures. He lay in a state of coma during forty-eight hours, from which (says Dr. L.) neither venesections (two in number) nor any other means could rouse him. Dr. Löwenhardt ordered two drachms of ung. hydr. to be rubbed in every two hours; and on the following day, ptyalism having been produced, consciousness began to return, and the patient eventually recovered.

Several cases of *acute hepatitis* are detailed, which were completely cured by mercurial inunction; all the symptoms giving way immediately on the appearance of salivation. The following is one of the cases:

A lady, thirty-six years old, of a delicate frame and weak constitution, was attacked by acute inflammation of the left lobe of the liver. Two days passed before the real nature of the affection was discovered; venesection was then had recourse to, a grain of calomel was given every two hours, and half an ounce of mercurial ointment was rubbed upon the abdomen daily. This treatment was persevered in for three days without any amendment following. Dr. Löwenhardt then discontinued every other remedy, and directed two drachms of ung. hydr. to be rubbed in every two hours; in less than forty-eight hours the gums began to be tender, and signs of amendment appeared, and perseverance for another day in this treatment was followed by complete restoration to health.

In recommending mercurial inunctions in *febris nervosa gastrica* (the *gastro-entérite* of the French writers), Dr. Löwenhardt is reviving the use of a remedy which was originally employed with great success in malignant fevers by Ambrose Paré, and has since been occasionally resorted to with good effect by some of the Italian physicians. He states that until very recently he was in the practice of treating this affection according to the rules usually laid down, but having observed that the mortality was almost always the same (about 1 in 7), whatever treatment was employed, or even when the cure of the patient was left almost entirely to nature, he was induced to search for some remedy likely to exert a more decided influence upon the course of the disease. In order fairly to test the value of mercurial inunction, no internal remedy was administered in those cases in which it was employed, though venesection or the application of leeches was premised when necessary; and when the disease took its usual course, the inunction was not commenced before the seventh or ninth day of the fever. In most cases, from two to three drachms of ung. hydr. were rubbed in every two hours, and the first symptoms of salivation usually made their appearance when from three to five ounces of the ointment had been used. A very evident diminution in the severity of the symptoms generally showed itself with the appearance of salivation, though in some cases, where the remedy had been resorted to late, the amendment was more gradual; all the patients, however, who were salivated recovered.

Of forty-eight persons thus treated, all were cured within from fourteen to twenty-one days; but one little girl, only seven years of age and of a sickly scrofulous habit, died nine weeks after her apparent recovery. Five other patients in whom cerebral affection predominated, and in whom the disease ran a very rapid course, died before salivation could be induced.

*Smallpox* is another disease on which Dr. Löwenhardt has tried the effect of mercurial inunction. He first sought by means of it to prevent the development of the smallpox pustules, and thus to cut short the disease; but in this he failed, and only succeeded in retarding the progress of the pustules by two or three days. He now proposed it merely as an occasional substitute for the cauterization of the pustules with nitrate of silver, which, though very efficient, is an exceedingly tedious process, and especially troublesome and difficult when children are the patients. After the pustules have been opened, he recommends their being smeared with ung. hydr., which, he assures us, produces the same effect of preventing pitting and cutting short the secondary fever, which follows the application of lunar caustic.

We have not room for a detail of Dr. Löwenhardt's experiments with regard to the nature of the eruption which appears after exposure to variolous infection, in those who have been previously vaccinated. They go to establish the fact that this eruption does not essentially differ from variola, and that smallpox is produced by inoculating with matter from its pustules.

The second memoir is devoted to a subject involved in considerable obscurity, namely, the periodicity of disease; it is entitled *On intermittent diseases, and in particular, on intermittent inflammations*, and opens with some observations on the difficulty of distinguishing inflammations with periodical exacerbations from intermittent fevers. Local inflammations, the daily exacerbations of which commence with rigour, are, in Dr. Löwenhardt's opinion, very often confounded with *quotidian intermittents;* and he thinks that practitioners just as frequently mistake the *febris gastrica subinflammatoria*, in which there exists an irritable or inflammatory condition of the glandular system or of the mucous membranes, for tertian intermittent. Several pages are devoted to the diagnosis between these two affections; but the points on which Dr. Löwenhardt most insists are the absence of those critical signs, the sweat and the deposit in the urine, which follow each paroxysm of real intermittent fevers, and the continuance of pain and uneasiness in the inflamed part during the intervals of the attack. In the treatment of these affections, though an antiphlogistic method is at first necessary, and local depletion is in many cases requisite, yet the subsequent employment of preparations of bark is often needed to complete the cure.

The third essay treats *Of the employment of mercurius vivus in introsusception.* The author considers that quicksilver has in these cases been far too hastily condemned by those who have had no personal experience of its effects; he regards it as a remedy well deserving a further trial; and in one of the cases which he relates, the preservation of the patient's life certainly appears to have been entirely owing to its administration. We shall give an abridgment of this case.

L———, a journeyman blacksmith, twenty-seven years old, a healthy man, but afflicted with a double inguinal hernia, as also with an abdominal hernia about two fingers breadth above the right groin, was attacked on the 1st of April, 1837, without any evident cause, with nausea, vomiting, pain in the abdomen, and tenesmus. It was first ascertained that there was no strangulation of intestine in any of the usual situations; purgatives were then administered, but no relief having been experienced, venesection was performed on the evening of the same day, and purgative enemata were given. The night was passed without relief; venesection was repeated and tobacco clysters were given on the following day. On the 3d, venesection was again performed and leeches were applied. The symptoms increased in severity during the 4th and 5th; the bowels could not be induced to act, feculent vomiting set in, and on the 6th the patient's recovery appeared impossible. As a last resource, Dr. Löwenhardt now administered three doses of quicksilver, an ounce each time, at intervals of one hour, followed by half-ounce doses of castor oil. At noon, immediately after taking the third dose, all the symptoms were much aggravated, the vomiting became very violent, and the castor oil was rejected, though the quicksilver remained on the stomach and the patient seemed to be dying. At four P.M., however, he experienced a sudden inclination to empty the rectum, and so violent a diarrhœa came on, that, within a very short time, the patient passed eighteen evacuations. The same evening he felt better; on the following day diarrhœa and a sense of weight in the abdomen were the only symptoms from which he suffered; and on the 9th, after having voided the quicksilver, he felt perfectly well, and has continued so ever since. (pp. 273-8.)

Want of space will not allow us to do more than to quote, in Dr. Löwenhardt's own words, the indications for the employment of this remedy; and we must refer our readers to the work itself for the history of several other cases, as well as for some interesting remarks on the diagnosis of internal strangulation (*incarceratio intestinorum interna*).

The cases in which the author conceives the administration of quicksilver to be justifiable are the following:

"1. *Volvulus.* Even in instances of the retrograde kind I cannot conceive that quicksilver should produce the inevitable fatal results which some have represented, although in such cases it would probably do no good; but there is no medicine of the unsuccessful employment of which we do not almost every day see some instance. Such is the case related by Willan, in which, though the quicksilver passed the bowels, it failed to relieve the *volvulus.*

"2. *Incarceratio interna.* In this case, if it should fail in producing its effect within twenty-four or thirty-six hours, it would be proper to have recourse to gastrotomy.

"3. *Ileus,* especially *ileus spasticus.* If the treatment, suggested by the nature of the exciting cause, should have proved unsuccessful.

"4. *Ileus inflammatorius.* If, after the symptoms which occur during the inflammatory stage have been subdued, the antiperistaltic motion of the intestinal canal should still continue, either from mere irritability or from exudation within the intestine.

"It is possible that, if the intestine were in part gangrenous, the quicksilver might cause laceration of its coats and extravasation into the abdominal cavity; but such cases would have a fatal result independently of the administration of quicksilver.

"5. If the symptoms of strangulation should continue after the performance of the operation for hernia, and reason should exist for supposing them owing to impermeability of the intestine from exudation upon its lining membrane, and agglutination of its walls.

"6. Lastly, violent vomiting, which other remedies have failed to relieve. Here I should employ it without hesitation, in the conviction that, at least, I could do no harm. Metallic mercury was also often employed with success, (*Panzoni, Description of a Disease which prevailed at Piarno, in Italy, in the year* 1786, *Lübben,*) during an epidemic, of which violent vomiting was one of the most prominent symptoms." (pp. 301-3.)

In the fourth essay—*On inflammation and caries of the lower jaw, produced by the appearance of the dens sapientis,* many novel and interesting facts are mentioned, relating to an affection hitherto overlooked or confounded with *cynanche parotidea.* Dr. Löwenhardt describes it as an inflammation of the *processus coronoideus* of the lower jaw-bone produced by the cutting of the wisdom tooth, and commonly attacking only the right side of the face. This inflammation soon extends to the ramus and sometimes to the body of the jaw, and terminates in the production of caries of the bone, having involved the soft parts at a very early stage: the parotid and submaxillary glands become inflamed and sometimes suppurate, and the severity of the disease in them often misleads the practitioner, inducing him to overlook that of the bone and to regard the case as one of simple inflammation of the salivary glands.

The remote causes of this affection are to be sought for, either in peculiarities in the form of the jaw-bone itself or in the arrangement of the teeth. Sometimes the body of the lower jaw is so small as to be completely filled up by the fourteen teeth which first appear, and no space is left for the *dentes sapientiæ;* in such a case they either remain completely within the jaw or, as sometimes happens, they take so great an inclination inwards as thus to avoid the crowding they would otherwise occasion. The last molar teeth in the lower jaw generally take more or less of an inward direction; but when that is not the case all the inconveniences of a small jaw-bone are much aggravated, and the cutting of the wisdom-tooth becomes a source of great irritation. In some cases, too, the coronoid process is found to deviate from its natural inclination even so far as to form an acute angle with the body of the jaw, while, at the same time, it loses its natural convexity outwards, and thus diminishes the space for the teeth in two directions. Where such a conformation of the jaw exists, the appearance of the wisdom-tooth produces the train of symptoms about to be described, the severity of which is, as might be expected, proportionate to the size of the crown of the tooth and the divergence of its roots.

The affection may be divided into two stages, the inflammatory and the suppurative. The first symptoms experienced by the patient are pain in the ear and in the articulation of the jaw on one side, commonly the right, and the movements of the jaw become constrained. The pain is increased towards evening or whenever the patient bites any hard substance; and it soon extends to the swollen parotid and submaxillary glands, and even to the muscles of the neck. After a time all the symptoms are aggravated, the swelling increases, deglutition is rendered difficult, the pain becomes more severe, and the part is so tender as not to bear the slightest touch. The patient continues with the neck stiff and the head turned somewhat to the affected side, for the slightest motion gives pain, and even becomes impossible as the disease proceeds. On examination of the mouth, the tonsil and the gum about the last molar tooth and about the articulation of the jaw of the affected side will be found red, swollen, and painful, and very often the top of the wisdom-tooth may be seen just making its appearance. By degrees the jaw becomes quite immoveable, so that the patient can take nothing but fluids; his general health suffers, and pain in the head, heat, thirst, and other febrile symptoms are produced by his sufferings. A sure sign that the caries of the bone has commenced, and that the disease has reached its second stage, is the loosening of the molar teeth of the affected side, which after a time can be removed without difficulty. Suppuration now takes place in the parotid and sometimes in the submaxillary gland; the matter burrows, forming sinuses among the muscles which sometimes are extensively destroyed, and at last escapes by various fistulous openings. This is followed by a diminution in the violence of the pain, the tension in the joint becomes less, and the patient finds himself able partly to open his mouth. When the disease has reached this height, the wound cannot heal until either nature or art has removed the dead bone; but if the case should fall under the eye of the practitioner at an earlier period, the extraction of the last molar tooth but one will often make room in the jaw, and remove the sufferings of the patient. Occasionally, however, the employment of leeches and of antiphlogistic treatment will also be necessary, and in some cases the practitioner is compelled to trust entirely to them, owing to the patient being unable to open his mouth. This treatment, however, leaving untouched the real source of the disease, is often insufficient, though it is frequently the only one adopted, owing to the practitioner confounding this affection with simple inflammation of the salivary glands, such as sometimes occurs sporadically in persons of a rheumatic constitution after exposure to wet; but it is very important that the practitioner should learn to distinguish between these two diseases.

This disease (*mandibulitis adultorum*) begins without any assignable cause: it attacks the right side of the face almost exclusively; its symptoms increase in severity in proportion to the duration of the affection, but the swelling of the glands is never very considerable, and at first deglutition is unaffected: the first stage lasts but for a short time, and with the commencement of the second stage the patient begins to recover the power of moving the jaw. Attacks of *angina rheumatica* can, on the contrary, be attributed to exposure to cold or to some similar cause; they usually begin acutely, and afterwards assume a more chronic character; they are accompanied by great swelling of the external parts, and deglutition is from the first much impeded; both sides of the face are affected, either at once or successively, and the disease has a great tendency to assume a chronic character, and be very tedious in its course.

The paper concludes with the history of several cases of this affection, in three of which partial amputation of the lower jaw was necessary.

Instead of quoting any of Dr. Löwenhardt's cases, we shall extract a similar one from an American journal, which shows that the disease has not been overlooked by our Transatlantic brethren. It is contained in a paper by Dr. Trudeau "On the varieties of deviation in the wisdom-teeth," originally published in the Medical Examiner, but taken by us from the Boston Medical and Surgical Journal for Aug. 1838.

"*The Wisdom-tooth arrested in its growth by the base of the coronoid process.* In this case the right cheek was swelled to an enormous size, the swelling extending from the eyes to the clavicle. The face and neck were covered with numerous abscesses. For twenty months the man had not been able to open his mouth, and had been fed on liquids, passed through an opening caused by the absence of a tooth. He had, besides, a fistula, at about three inches from the lower angle of the inferior maxilla; a little lower on the neck there was another. A probe being introduced into the first fistula, penetrated obliquely from before backwards, and was stopped by a hard body, supposed to be the root of the third large grinder. From the beginning of the affection, the man's health had been greatly impaired; he was much emaciated, his skin was of a leaden hue, and he suffered much from colics. For a short time past, his digestion had been disordered and attended with acidity; this, probably, depended on the mixture of his food with the pus, with which his mouth was constantly filled. Various means were resorted to, to open the patient's mouth and extract the tooth. Leeches, poultices, mercurial frictions, blisters, and compression were used with no better success. Dr. ——— now thought of trying a mechanical agent, which succeeded perfectly. It was a conoidal piece of wood, introduced between the dental arch, and pushed in slowly by the patient himself. The following day the mouth presented an opening of about four lines. A week afterwards, the man's mouth was opened wide enough to allow of the tooth being easily extracted. A few days after, a necrosed piece of bone was extracted. It proved to be a portion of the base of the coronoid process, on which was moulded or cast a portion of the crown of the tooth. This evidently showed that the tooth had been stopped in its growth by this bone. Since that time the inflammation rapidly disappeared, and in a month the patient was perfectly cured."

Besides the above essays, the work contains a few short memoirs upon obstetric subjects, which we have not now space to allude to, but accounts of which we may hereafter lay before our readers.

II. Dr. Jahn is also known to our readers as the author of a singular work on Pathology reviewed in our Seventh Number. The present is a collection of short treatises on various subjects connected with practical medicine, on which it has been the author's object to throw additional light. His observations are valuable, and are exceedingly interesting, from the enthusiasm and learning stamped on almost every page, and from the many bright suggestions with which they abound. In perusing them we are struck with surprise to find no allusion made in any part to the author's novel system of pathology, or *physiatrics*, as he has termed it, noticed at full length in our Seventh Number. The present volume is of a totally different character, and its chief aim is to communicate practical knowledge. Theory, however, is not altogether neglected, but facts and cases are detailed in general simply as they occurred; and when hypothesis is added, it does not detract from the value of the observations on which it is founded.

1. The first and most important article is upon acute hydrocephalus. During the spring of 1834 a very fatal epidemic occurred at Meiningen, and in a space of seven or eight weeks, twenty-one cases came under Dr. Jahn's notice, of which twelve proved fatal. Our author enters very fully upon the nature and treatment of the disease, and details at considerable length the appearances observed after death, analyzing and criticising at the same time the opinions of other authors. He urges the practitioner to continue his exertions to save his patient even after indubitable signs of effusion have shown themselves, and relates four cases of complete recovery after transudation had taken place. In two of these, the favorable termination appeared to be owing to the application of large blisters to the nape of the neck and scalp, and in the other two, to the almost unaided efforts of nature. There exists, we are aware, very often considerable difficulty in determining whether effusion has or has not taken place, and many physicians, and among others Gölis,* have expressly stated their opinion that this state, when once it has occurred, precludes all hope of recovery. The first two cases detailed by Jahn are given so fully that they have left the impression on our mind that in them effusion really had taken place, but in the latter two it is merely stated, that transudation having ensued, the case was considered hopeless and given up by the physician, and that nevertheless the children recovered. A case, we hold, should never be given up; we have seen cases of hydrocephalus given up both in public hospitals and in private practice; and although death invariably ensued in such cases, we are not warranted in assuming that such would have been the result, had some energetic means been resorted to.

2. In several cases of paralysis, more or less complete, dependent on spinal affections, Dr. Jahn had recourse to the spirituous extract of nux vomica, but in none of the cases did he derive any benefit from its use. Indeed, in his hands the medicine seems to have been totally inert. In one case he carried the dose to twelve grains daily, in another to seventeen, in a third to twenty, and in a fourth to thirty grains daily, but in none of these cases had it any effect in diminishing the paralysis, nor does it in any way affect the nervous system. We cannot help suspecting that the extract used by Dr. Jahn had either been intentionally adulterated, or had suffered in some way by long keeping.

3. The ninth article is one of considerable length, and is devoted to the consideration of the secondary symptoms of gonorrhœa. This has long formed one of the vexed questions of medicine, and is one on which those who have the best means of coming to a decision are still not agreed. Ricord denies in the strongest terms that secondary symptoms ever follow on gonorrhœa unconnected with chancre, whilst another surgeon, Cullerier,† enjoying the same opportunities of study, freely admits

* Von der hitzigen Gehirnholenwassersucht. Band i. p. 195, et seq.

† Dict. de Méd. et de Chir. prat. Art. Blennorrhagie et Syphilis.

the possibility of general lues from simple gonorrhœa. But Jahn, in accordance with the opinion of several German physicians, among whom we may mention Autenrieth and Schönlein, believes that gonorrhœa is occasionally followed by secondary symptoms peculiar to itself, and different in nature from those which follow on chancre. The catalogue of evils which Dr. Jahn enumerates as liable to follow upon gonorrhœa is quite appalling, but we must do him the justice to say that he acknowledges never having met with them in his own practice. He mentions acute and chronic inflammation of the mucous membranes of the air-passages and intestines, stricture of the œsophagus, scirrhus of the pylorus, excrescences on the intestinal mucous surface, ulcers on the skin, inflammation of the joints, condylomata of the heart, tubercles in the various glands, and many other affections of equal importance. Dr. Jahn relates three cases of gonorrhœa which lately came under his notice and which were followed by secondary symptoms, but these showed nothing different from the common symptoms of secondary syphilis; and five cases of chronic disease which he cites at the end of the article, in illustration of the doctrines contained in it, afford no reason for supposing that they were a consequence of gonorrhœa. It is maintained by some surgeons that secondary symptoms, which make their appearance after an apparent simple gonorrhœa, must owe their origin to a chancre which had escaped notice by being situated in the urethra, or from some other cause, whilst others contend that secondary symptoms may follow upon what they term syphilitic gonorrhœa; that is, upon an inflammation of the mucous membrane dependent on syphilitic contagion. We are not aware, however, that any distinction has ever been drawn in this country between the symptoms which follow on the one affection or on the other, so as to mark them as two distinct diseases, and there are no arguments in Dr. Jahn's paper which tend in any way to convince us that such is the case. The paper displays great erudition, but it possesses little practical value; and Dr. Jahn does not detail a single case as having occurred to himself with which he could fortify his hypothesis.

4. In the seventeenth paper the author notices the results of an investigation which he conducted with occasional interruptions during a space of two years, in order to satisfy himself of the truth or falsity of the homœopathic doctrines. He was led by these experiments to put faith, if not in the infinitesimally small doses of the homœopathists, at least in doses small in comparison to those to which he had been accustomed. Goitre is an endemic and frequent disease in the neighbourhood of Meiningen, and experience has amply proved that it yields only to iodine, burnt sponge, and similar remedies. It was therefore with surprise that Dr. Jahn saw the swelled thyroid gradually diminish in size under the use of powders, containing each $\frac{1}{60}$th of a grain of iodine, administered either daily or every second day. Thirteen cases of goitre were thus treated; fourteen days was the shortest space, and five weeks the longest space required to effect the cure. Smaller doses he found inefficacious. Our present space does not allow us to notice any of the other papers particularly, but we can recommend them as containing much interesting and instructive matter.

